# Supplementary material for: Strongly Metal-Adhesive and Self-Healing Gelatin@Polydopamine-Based Hydrogels with Long-Term Antioxidant Activity
Source: Antioxidants (Basel). 2023 Sep 14;12(9):1764. doi: 10.3390/antiox12091764 (PMC10525539; doi:10.3390/antiox12091764)
Supplement: Supplementary file 1 [file antioxidants-12-01764-s001.zip › antioxidants-2601502-supplementary.pdf]

Strongly metal adhesive and self-healing gelatin@polydopamine based hydrogels with long term antioxidant activity

Jordana Hirtzel [1,2], Guillaume Leks [1,3], Julie Favre[1], Benoît Frisch [1], Isabelle Talon [3,4], Vincent Ball [2,3,*]

[1] 3Bio, Laboratoire de Conception et Application de Molécules Bioactives, UMR 7199. Université de Strasbourg/CNRS. Faculté de Pharmacie, 74 route du Rhin, Illkirch F-67401 Cedex, France; frisch@unistra.fr

[2] Faculté de Chirurgie Dentaire. Université de Strasbourg. 8 rue Sainte Elizabeth, Strasbourg 67000, France; vball@unistra.fr

[3] Biomatériaux & Bioingénierie, UMR_S 1121. Université de Strasbourg/INSERM. 1 rue Eugène Boeckel, Strasbourg 67000, France; vball@unistra.fr

[4] Hôpitaux Universitaire de Strasbourg, Service de Chirurgie Pédiatrique. Strasbourg, France; isabelle.talon@chru-strasbourg.fr

* Correspondance: vball@unistra.fr

Supporting Information

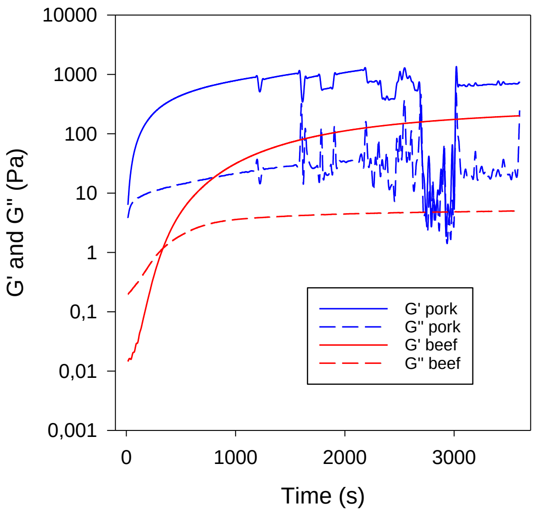

Figure S1. Gelation kinetics of PDA-gelatin gels followed through rheology, for porcine (in blue) and bovine (in red) gelatin. The solid curves represent G′ while dashed ones correspond to G′′ (Pa). Data were taken from three unrelated experiment each.

Instabilities after 1000 s for pork gelatin are systematic and due either to some sliding movement of the gel between the cone and the plate of the rheometer or to the formation of self-healing cracks in the gel.

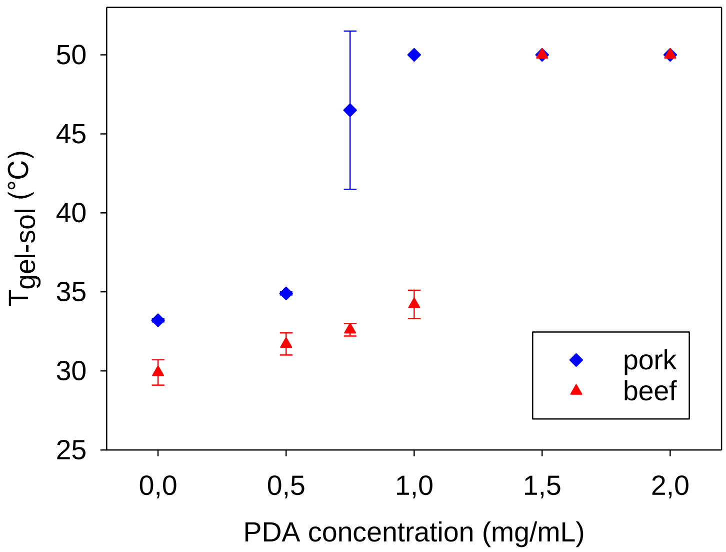

Figure S2. Gel-sol temperature depending on the PDA concentration of PDA-gelatin gels measured by rheology for porcine (◆) and bovine (▲) gelatin. If the gel-sol temperature is not reached at 50 °C, the point is set to 50 °C, which is the maximum temperature measurable with the used rheometer. Data and standard deviation come from three unrelated experiments.

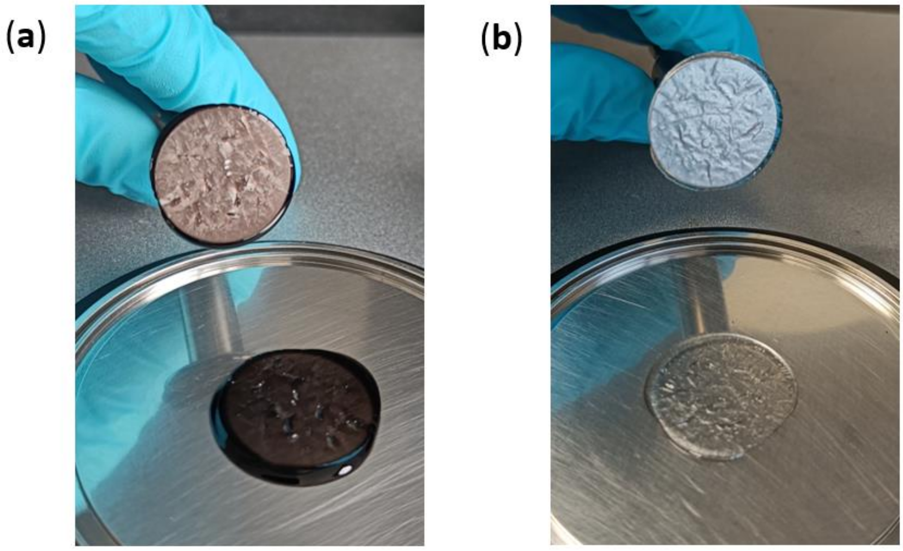

Figure S3. Photographs of the cohesive nature of the hydrogels: (**a**) with PDA; (**b**) without PDA.

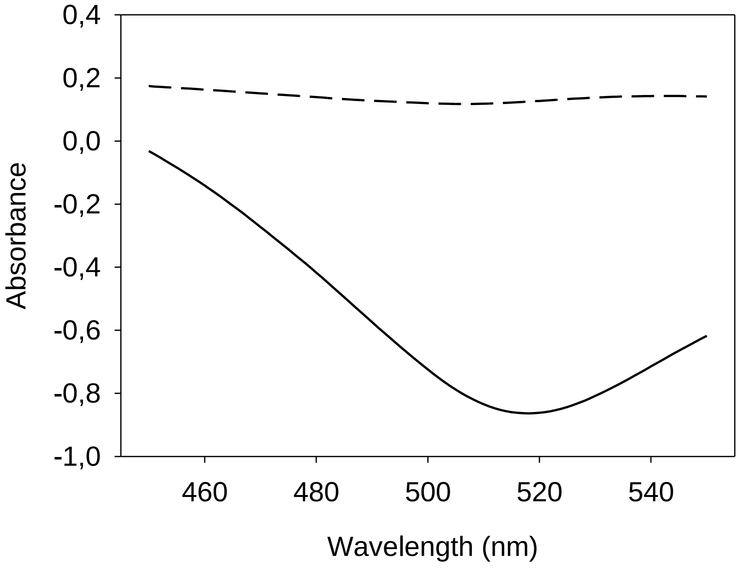

Figure S4. Antioxidant properties of gelatin gels. Absorbance spectra of a DPPH solution in contact with a 2 mL gel after 4 h, with the DPPH control solution as reference. The solid curve corresponds to PDA-gelatin gel, while the dashed curve, the pristine gelatin gel.

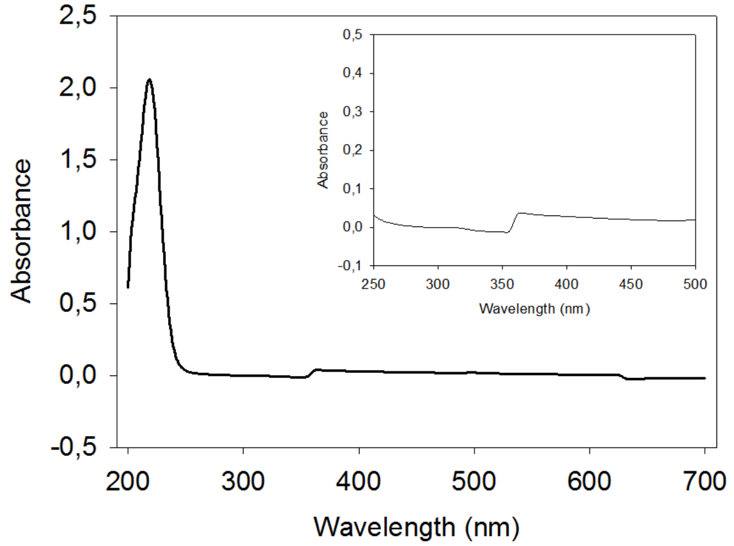

Figure S5. UV-visible spectra of the ethanol supernatant after immersion of 2 mL of PDA-gelatin gel in 30 mL absolute EtOH for 48 h. The release was performed in conditions of total darkness. The inset corresponds to a magnification in the wavelength interval between 250 and 500 nm. The kink at 360 nm corresponds to the shift from the deuterium lamp to the mercury vapor lamp of the spectrophotometer.
